# Radial nerve risk in bicortical implant trajectories for pediatric medial epicondyle fixation: A 3D MRI-based observational study

**DOI:** 10.1097/MD.0000000000043944

**Published:** 2025-08-22

**Authors:** John P. Avendano, Myung-Jin Cha, William ElNemer, Christa L. LiBrizzi, Shivani Ahlawat, Rushyuan Jay Lee

**Affiliations:** a Department of Orthopaedic Surgery, Johns Hopkins Hospital, Baltimore, MD; b Department of Radiology, Johns Hopkins Hospital, Baltimore, MD.

**Keywords:** ideal trajectory, medial epicondyle fracture, pediatric medial epicondyle fracture fixation, radial nerve, screw fixation

## Abstract

Distal humeral medial epicondyle avulsion fractures are commonly stabilized with implants directed proximally and anterolaterally. In this cohort study, we evaluated the location of the radial nerve relative to an ideal bicortical implant for pediatric medial epicondyle fractures. We aimed to (1) characterize the location of the radial nerve relative to a simulated ideal bicortical wire or screw trajectory for medial epicondyle fracture fixation and (2) recommend a safe implant trajectory. We identified 40 patients (40 arms; 19 male, 21 female) aged 7 to 16 years with 3-dimensional (3D) upper extremity magnetic resonance images done from September 2018 to 2023. Exclusion criteria were absence of 3D sequences, fused apophyses, and fractures or tumors. Multiplanar reformations were generated from 3D sequences and an ideal distal humeral implant trajectory was simulated. The optimal implant crossed centrally and perpendicular to the medial epicondyle apophysis and passed superior to the olecranon fossa. Outcome variables were (1) angle between implant trajectory to radial nerve; (2) anteroposterior distance from implant trajectory to radial nerve; and (3) “overshoot” distance from lateral humeral cortex implant exit to radial nerve. Descriptive statistics are reported. Of 208 consecutive elbow magnetic resonance imaging, 40 patients (19 male, 21 female) with a median age of 13 years (range: 8–15 years) met inclusion criteria. The angle between an optimal implant trajectory to radial nerve, when measured from the medial epicondylar entry point, was a mean 8.7 degrees ± 3.2 (range: 4.3–20.4 degrees). The mean anteroposterior distance was 0.8 cm ± 0.3 (range: 0.3–1.6 cm). The mean “overshoot” distance was 0.98 cm ± 0.44 (range: 0.32–2.37 cm). In 30 patients, the radial nerve was anterior to our ideal implant trajectory; in 10 the radial nerve was posterior. Given the proximity of the radial nerve (<1 cm from lateral humeral cortex implant exit) and the variability of its position (anterior vs posterior relative to an ideal implant trajectory), ascertaining a universal safe trajectory for bicortical implant placement for fixation of medial epicondyle avulsion fractures is not possible.

**Level of Evidence:**

IV.

## 1. Introduction

Medial epicondyle avulsion fractures commonly occur in children, most often in boys aged 9 to 14 years, and are increasing in frequency secondary to an increasingly athletic pediatric population.^[1]^ They account for 11% to 20% of all pediatric elbow fractures, and can occur because of *pull-off* injuries caused by valgus stress at the elbow and contractions of the flexor muscles.^[1–4]^ Whereas both nonoperative and operative management can address these injuries, operative management, which is often considered for markedly displaced fractures in athletes who require valgus stability of the elbow, typically consists of open reduction and internal fixation with the use of a wire or screws.^[5,6]^ These implants often traverse proximally in the distal humerus in a posterior-to-anterior direction.^[7]^

The ideal implant, a screw or one wire in a wire construct that stabilizes these fractures, is often inserted perpendicular to the medial epicondyle apophysis, directed proximally and posteromedially to anterolaterally. Although the ulnar nerve is anatomically closest to the medial epicondyle and implant insertion site, there have been instances of radial nerve injury secondary to medial epicondyle fracture fixation.^[8]^ Despite the known risk of damage to the ulnar nerve and attempts to protect it during distal humeral implant insertion, rates of iatrogenic ulnar nerve injury ranging from 0.4% to 15% for children being treated for supracondylar humerus fractures, hovering around 2% for children being treated for medial epicondyle fractures, have been reported.^[6,9,10]^ In contrast, to our knowledge, no source has estimated the true frequency of iatrogenic radial nerve palsies secondary to distal humeral implant insertion; the available literature consists mainly of case series and anatomic studies discussing its overall course.^[11–15]^ Safe zones to avoid iatrogenic nerve injury in approaching the pediatric distal humerus have been described,^[11,13]^ yet no study has explicitly discussed anatomy directly pertinent to medial epicondyle avulsion fracture fixation.

As such, the purpose of this study was to evaluate the anatomic relationship between the radial nerve and a simulated ideal bicortical implant trajectory on 3-dimensional (3D) magnetic resonance imaging (MRI) of native, uninjured pediatric elbows. This modeling approach was designed to simulate a “best-case” implant path through normal, unfused epicondylar anatomy, minimizing confounding by fracture morphology, displacement, or hardware.

Our aim was to (1) characterize the location of the radial nerve relative to an ideally placed bicortical implant among pediatric patients with normal distal humerus anatomy; and (2) assess whether a universally safe implant trajectory can be identified based on these simulations. This information may help inform implant planning for medial epicondyle avulsion fractures by defining the anatomic proximity of the radial nerve in the absence of fracture distortion and by identifying “high-risk” angular corridors for radial nerve injury. Ultimately, we sought to provide parameters for safer implant placement by establishing normative anatomic relationships using high-resolution 3D MRI data.

## 2. Methods

This Health Insurance Portability and Accountability Act-compliant, retrospective observational study used de-identified MRI data and was therefore granted exempt status by The Johns Hopkins Medicine Institutional Review Boards (IRB #00401256). The study involved pediatric patients undergoing 3D MRI of the elbow from 09/2018 to 09/2023 at our hospital.

### 2.1. Study population

We retrospectively identified, on our picture archiving and communication system, consecutive MRIs of the elbow of children between the ages of 7 and 16 years taken from September 2018 to September 2023. Patients were excluded if they had any type of fracture involving the distal humerus, including but not limited to medial epicondyle fractures, to ensure that native anatomic relationships were preserved and not altered by displacement, periosteal reaction, or callus formation. Because of the advanced imaging requirements for 3D multiplanar reconstruction and the rarity of high-quality, native MRIs in skeletally immature patients without fused apophyses, our final cohort size reflects the strict inclusion criteria necessary for accurate anatomic modeling.

### 2.2. 3D MRI acquisition

Volumetric 3D MRI sequences included sagittal proton density ([PD] [repetition time: 1000 milliseconds (ms); echo time: 28 ms; voxel size: 0.5 × 0.5 × 0.5 mm]) and T2-fat suppressed ([FS] [repetition time: 950 ms; echo time: 108 ms; voxel size: 0.6 × 0.6 × 0.6 mm]) performed without the administration of intravenous contrast material on a 3.0 Tesla Skyra™ (Siemens Healthineers, Erlangen, Germany) magnet system with a field of view of 16 × 24 cm.

### 2.3. Anatomic measurements

**Figure 1.**
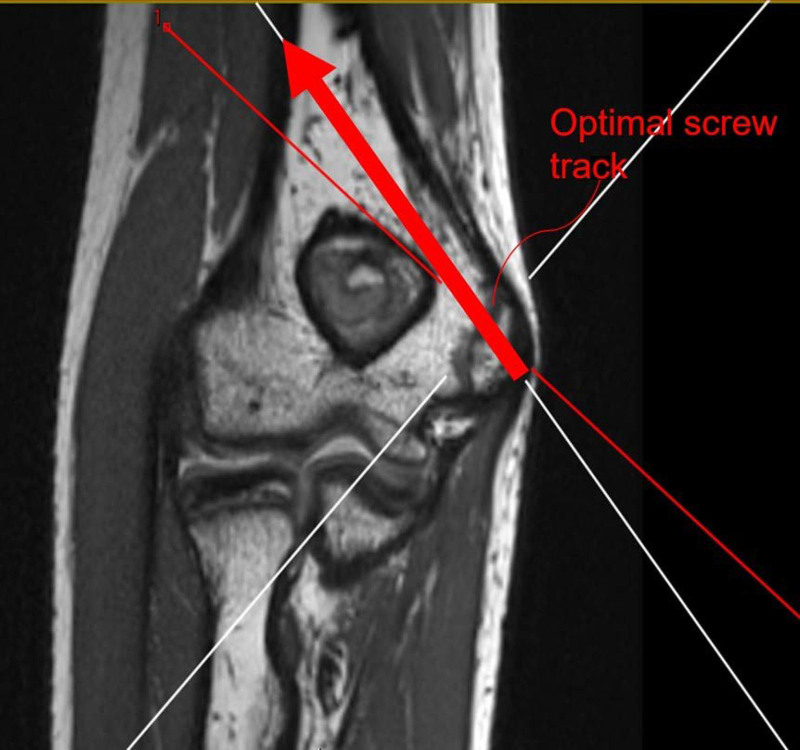
Coronal view of MRI displaying ideal implant track with entry near perpendicular to the medial epicondyle and cranial to the olecranon fossa. MRI = magnetic resonance imaging.

**Figure 2.**
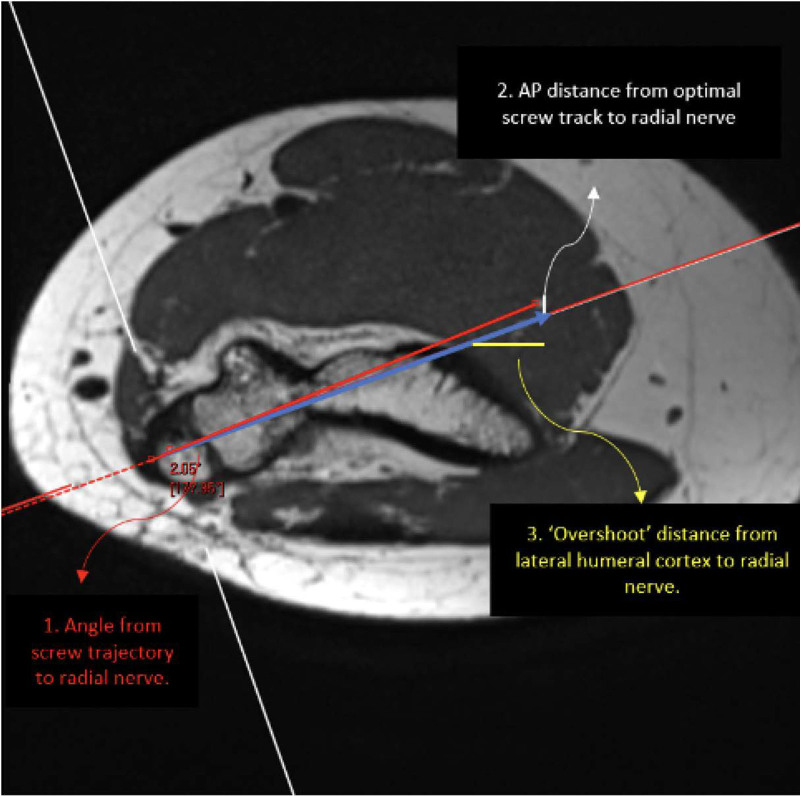
Axial oblique MPR image displaying “optimal” implant track with perpendicular entry to medial epicondyle, implant positioned medially to laterally, and various distances of interest labeled. AP = anterior–posterior; MPR = multiplanar reformation.

First, sagittal PD non-FS was selected and converted to axial, sagittal, and coronal multiplanar reformations. Second, an arrow simulating the ideal implant was placed on the coronal image perpendicular to the medial epicondyle apophysis and adjusted to pass cranial to the olecranon fossa (Fig. 1). Third, the ideal implant trajectory was identified on the axial image, also perpendicular to the medial epicondyle apophysis, and an axial oblique image was generated (Fig. 2). Lastly, the radial nerve was identified, and various measurements were made relative to the ideal implant trajectory. This trajectory was determined in consultation with a pediatric orthopedic surgeon to reflect standard surgical technique. On picture archiving and communication system, which had built-in calibrated tools for measurement, a single reader performed all measurements. Outcome variables were (1) the angle between the ideal implant’s trajectory tip at the lateral humeral cortex to the radial nerve, (2) the anterior-to-posterior distance from the ideal implant to the radial nerve, and (3) the “overshoot” distance from the lateral humeral cortex to the radial nerve. The anatomic location, anterior or posterior to the ideal implant trajectory, was recorded. Measurements using the same technique were repeated on 3D T2FS. Rather than fixed distances, anatomic landmarks were used to assess angular measurements.

### 2.4. Statistical analysis

Descriptive statistics characterized the variance of the radial nerve and its location in our patient population. All analyses were performed with R 4.4.0 (R Core Team, 2024). To assess whether the imaging sequence (3D PD vs 3D T2FS) affected measurements, independent samples *t* tests were used to compare mean values between groups. A *P* value < .05 was considered statistically significant. The statistics we included were evaluated by an individual with expertise in statistics.

## 3. Results

**Figure 3.**
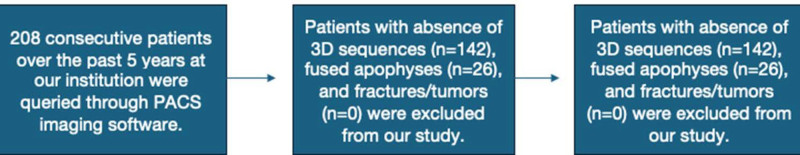
Flowchart detailing application of exclusion and inclusion criteria to patients. PACS = picture archiving and communication system.

Our search yielded 208 MRIs. MRIs were excluded because of absence of 3D sequences (n = 142), having fused epicondylar apophyses (n = 26), having fractures (n = 0), having tumors (n = 0), and having instrumentation (e.g., screws, plates, or wires in the distal humerus) (n = 0). The final cohort was 40 patients (median age 13 years, range: 8–15 years; 19 boys, 21 girls). The application of elbow inclusion and exclusion criteria is explained in the flowchart below (Fig. 3).

### 3.1. Anatomic variation of the radial nerve

The mean angle between our “ideal” distal humeral bicortical implant and the radial nerve was 8.7 degrees with a standard deviation of 3.2 degrees (range: 4.3–20.4) on 3D PD. The mean anteroposterior (AP) distance from our optimal bicortical implant to the radial nerve was 0.8 cm ± 0.3 (range: 0.3–1.6 cm) on 3D PD. The mean overshoot distance from our optimal bicortical implant to the radial nerve was 1.0 cm ± 0.4 (range: 0.3–2.4) on 3D PD. There were no significant differences in the measurements between 2 3D sequences (PD vs T2FS). Distribution of the measurements is shown in Table 1.

**Table 1:** Distribution of mean overall angles and distances relative to our “ideally” placed implant.

Variable	Mean ± standard deviation		*P*-value
	3D PD	3D T2FS	
Angle between optimal distal humeral implant to radial nerve (degrees)	8.7 ± 3.2	8.3 ± 3.1	.5
AP distance (cm)	0.8 ± 0.3	0.8 + 0.2	.7
“Overshoot” distance (cm)	1.0 ± 0.4	0.9 + 0.4	.4

3D PD = 3-dimensional proton-density, 3D T2FS = 3-dimensional T2 fat-suppressed, AP = anterior–posterior.

### 3.2. Safe implant trajectory

Although we recorded measurements in 40 separate elbows, the variance of the radial nerve was noteworthy. In 30 of our patients, the radial nerve was anterior to our ideal bicortical implant, whereas in 10 of our patients, it was posterior to the ideal bicortical implant. When anterior, the mean angle between our ideal distal humeral implant and the radial nerve was 8.6 degrees ± 3.4, whereas when posterior, our mean angle was 9.0 degrees ± 3.1 (Table 2).

**Table 2:** Distribution of average overall angles and distances relative to our “ideally” placed implant when separated by radial nerve location.

Anatomic location relative to the screw trajectory	Anterior (n = 30, 75%)	Posterior (n = 10, 25%)
Angle between optimal distal humeral implant to radial nerve (degrees)	8.6 ± 3.4	9.1 ± 3.1
AP distance (cm)	0.7 ± 0.3	0.8 ± 0.3
“Overshoot” distance (cm)	1.0 ± 0.5	1.0 ± 0.3

AP = anterior–posterior.

## 4. Discussion

Given the proximity of the radial nerve (<1 cm from lateral humeral cortex implant exit and ~9 degrees anterior or posterior to ideal implant trajectory’s perpendicular entry to the medial epicondyle) and the variability of its position (anterior vs posterior relative to an ideal implant trajectory), a universal clinically applicable safe trajectory for bicortical implant placement for fixation of medial epicondyle avulsion fractures is not possible. Further, although there is typically a buffer from the far cortex to the nerve (mean 1.0 cm), the radial nerve sometimes abuts the cortex (3 mm). Knowledge of the radial nerve’s anatomic variation and close proximity during medial epicondylar fracture fixation is of critical importance to avoid iatrogenic injury upon insertion of wires or screws. This knowledge is helpful for informing surgeons of the potential risks that may come with what is considered a relatively straightforward and complication-free procedure.^[6,16]^

Previous studies have documented landmarks of which to be wary when localizing the radial nerve, as well as “safe zones” of entry for pinning in the distal humerus in children. None, however, has explicitly discussed these parameters in the context of medial epicondyle fracture fixation. In a cadaveric study evaluating the relative location of the brachial portion of the radial nerve with regard to surgical implications, Artico et al^[17]^ found that the mean distances between the point where the radial nerve crosses the lateral border of the humeral shaft and the lateral and medial epicondyles were 125 (±13) and 129 (±13) mm, respectively.^[15]^ Theeuwes et al^[15]^ also performed a cadaveric study and found that the distance from the medial epicondyle to the point where the radial nerve bends posteriorly to laterally was 142 mm on an AP radiograph and 152 mm on a lateral radiograph; the mean distance from the medial epicondyle to the point where the radial nerve bends laterally to anteriorly was 66 mm on an AP radiograph; and that on a lateral radiograph, the nerve moves away from the anterior cortex by 83 mm, positioning itself toward the center of the capitellum and trochlea.^[15]^ Though both of the aforementioned studies used adult cadavers, they also demonstrated variability in crossing of the radial nerve from posterior to anterior. This variability in adults supports our study finding: that the position of the radial nerve relative to bicortical medial epicondyle implants in pediatric patients is also variable.

Bloom et al^[11]^ utilized previous 2-dimensional MRIs and elbow radiographs in 23 pediatric patients to define a straightforward parameter for the superolateral approach to distal humerus fractures, ultimately finding the radial nerve to be consistently located > 20 degrees anterolateral to the transepicondylar axis in the axial plane. This culminated in the development of the lateral supracondylar ridge line method, a straightforward and safe parameter that can be applied to pediatric patients intraoperatively.^[11]^ This method informs surgeons that, in children, hardware can be safely inserted into the distal humerus along the transepicondylar axis, either at or slightly posterior to the lateral supracondylar ridge, when placed caudal to the point where the lateral supracondylar ridge line diverges from the proximal extent of the supracondylar ridge on an AP elbow radiograph.^[11]^ Although this method may be helpful for the lateral approach to the distal humerus, it is not applicable to medial epicondyle fracture fixation. Furthermore, although the radial nerve tends to be anterolateral to the transepicondylar axis in children, it can be anterior to posterior to the medial epicondyle bicortical implant trajectory, suggesting that the implant trajectory and transepicondylar axis are not interchangeable.

Previous studies have purported that the position of the radial nerve maintains a linear relationship with arm length in growing children and is thus more or less predictable with regard to palpable osseous landmarks.^[13]^ Nonetheless, it is clear from our results that its location can be variable relative to implant trajectories in children. In fact, studies have shown that upper extremity neurovascular structures in the pediatric population are not consistently traceable in their anatomic courses, which can lead to unanticipated neurovascular injuries.^[13,14]^ As such, it is important to know that the relationships between neurovascular structures and osseous landmarks in the pediatric population remain ambiguous. Future cadaveric and imaging studies should be done to better elucidate the various courses of these structures in the growing child.

Our study has several limitations, including small sample size (n = 40); retrospective, single-center design; and wide age range/skeletal maturation of the included cohort, all of which may affect the generalizability of these results. Measurements were made on 3D elbow MRIs that were of varied image quality, and because of the small size of the radial nerve on axial oblique MRIs, the precision of our measurements may not be perfect; however, they are likely within 1 to 2 mm. Unlike 2D MRI sequences, inclusion of 3D isotropic volumetric sequences enabled multiplanar generation with highest spatial resolution possible. Our limited patient sample size may over- or underestimate the true level of variance in radial nerve anatomy in the pediatric elbow. Our study’s limited sample size reflects the relative rarity of pediatric elbow MRIs that both include 3D multiplanar reformation sequences and meet strict criteria for normal, unfused medial epicondylar anatomy. Nonetheless, the high fidelity of our dataset supports the reliability of the observed anatomic relationships. Overall, our study, to our knowledge, is the most comprehensive anatomic study of the radial nerve in the pediatric population and the only one to discuss radial nerve anatomy in the context of medial epicondyle fracture fixation.

## 5. Conclusion

Our study provides an understanding of the variable relative position of the radial nerve in pediatric elbows. Although our study aim was to detail a safe trajectory for bicortical fixation of medial epicondyle elbow fractures, given the variance of the radial nerve, we cannot propose a safe or identifiable path that consistently avoids iatrogenic radial nerve injury. Minor encroachment into the soft tissues when drilling through the far lateral cortex from the medial epicondyle of the elbow can injure the radial nerve. Protective measures to avoid radial nerve violation during surgical treatment of the medial epicondyle should focus on unicortical implant constructs or surgical techniques that minimize intrusion of drills and implants beyond the lateral humeral cortex. Although our data highlight consistent proximity of the radial nerve to the ideal trajectory in certain angular corridors, further studies with larger populations are warranted to confirm these trends and refine safe zone recommendations.

## Acknowledgments

For editorial assistance, we thank Denise Di Salvo, MS, who provided permission to be acknowledged, in the Editorial Services group of The Johns Hopkins Department of Orthopaedic Surgery.

## Author contributions

**Conceptualization:** John P. Avendano, Myung-Jin Cha, William ElNemer, Rushyuan Jay Lee.

**Formal analysis:** John P. Avendano, Myung-Jin Cha, William ElNemer.

**Writing – original draft:** John P. Avendano, Myung-Jin Cha, William ElNemer, Christa L. LiBrizzi, Shivani Ahlawat, Rushyuan Jay Lee.

**Writing – review & editing:** John P. Avendano, William ElNemer, Christa L. LiBrizzi, Shivani Ahlawat, Rushyuan Jay Lee.
